# Comparison of the adherence of nontypeable *haemophilus influenzae* to lung epithelial cells

**DOI:** 10.1186/s12879-024-09085-7

**Published:** 2024-02-12

**Authors:** Yuwei Rong, Zihao Liu, Heping Wang, Zuguo Zhao

**Affiliations:** 1 Shantou University Medicine College, Shantou University (STU), Shantou, Guangdong 515041 China; 2 Shenzhen Children’s Hospital, Shenzhen, Guangdong 518038 China; 3 Department of Microbiology, Immunology of Basical Medicine of Guangdong Medical University, Dongguan, Guangdong 523810 China

**Keywords:** *Nontypeable haemophilus influenzae* (NTHi), Children, Adherence, A549 cells

## Abstract

**Objective:**

*Nontypeable Haemophilus influenzae* (NTHi) plays an important role in respiratory tract infections, and adherence to lung epithelial cells is the first step in lung infections. To explore the role of NTHi in childhood lung infections, a comparative study was conducted on the adherence of strains isolated from sputum culture and bronchoalveolar lavage fluid to A549 lung epithelial cells.

**Methods:**

*Haemophilus influenzae* strains were obtained from the sample bank of Shenzhen Children’s Hospital, and identified as NTHi via PCR detection of the capsule gene *bex*A. NTHi obtained from healthy children’s nasopharyngeal swabs culture were selected as the control group, and a comparative study was conducted on the adherence of strains isolated from sputum culture or bronchoalveolar lavage fluid of patients to A549 cells.

**Results:**

The adherence bacterial counts of NTHi isolated from the nasopharyngeal cultures of healthy children to A549 cells was 58.2 CFU. In patients with lung diseases, NTHi isolated from bronchoalveolar lavage fluid was 104.3 CFU, and from sputum cultures was 115.1 CFU, both of which were significantly higher in their adherence to A549 cells compared to the strains isolated from the healthy control group. There was no significant difference in adherence between the strains isolated from sputum cultures and bronchoalveolar lavage fluid (t = 0.5217, *p* = 0.6033).

**Conclusion:**

NTHi played an important role in childhood pulmonary infections by enhancing its adherence to lung epithelial cells.

**Supplementary Information:**

The online version contains supplementary material available at 10.1186/s12879-024-09085-7.

## Introduction

*Haemophilus influenzae* is a common opportunistic pathogen that is often isolated from the upper and lower respiratory tracts [1, 2]. The widespread vaccination of *Haemophilus influenzae* B conjugate vaccine in the domestic pediatric population has made *nontypeable Haemophilus influenzae* (NTHi) the main pathogenic bacteria causing respiratory tract infections in children [3]. NTHi colonizes the human nasopharynx, but is an important pathogen in middle ear infection (otitis media) in children [4] and exacerbations in bacterial bronchitis, chronic obstructive pulmonary disease, and bronchiectasis [5, 6], as well as community-acquired pneumonia, in adults [7]. Frequency of disease caused by NTHi is increasing annually, exacerbated by both the absence of an NTHi vaccine and emerging antibiotic resistance [8]. In the process of pulmonary infection, adhesion to pulmonary epithelial cells is the first step of pulmonary infection [6, 9, 10], which directly affects its pathogenic ability. Many studies have been conducted on cell adhesion and related factors in otitis media and adult chronic obstructive pulmonary disease [5, 6, 11], but there are few studies on the adhesion of epithelial cells in children with pulmonary infections, such as pneumonia, bronchiectasis, persistent bacterial bronchitis, and chronic suppurative lung disease. In this study, *Haemophilus influenzae* isolates were selected from the nasopharynx of healthy children and from the sputum or bronchoalveolar lavage fluids of hospitalized children. The adhesive abilities of these isolates to A549 cells were compared in vitro, to explore the mechanism of *Haemophilus influenzae* infection in the progress of pediatric pulmonary infections.

## Materials and methods

### *Haemophilus influenzae* strains

From the sample bank of Shenzhen Children’s Hospital, *Haemophilus influenzae* were selected from samples collected during the period from June 2019 to May 2022. These *Haemophilus influenzae* were cultured from the nasopharynx of healthy children and from the sputum or bronchoalveolar lavage fluids of hospitalized children with pulmonary infections, including both acute and chronic infections. Inclusion criteria for pulmonary infection group: ① Age range from 1 month to 14 years old; ② Meeting the diagnostic criteria for pulmonary infection [8], infections in the lung parenchyma and/or interstitial areas can cause varying degrees of hypoxia and infection symptoms, usually accompanied by fever, cough, increased breathing, and moist rales in the lungs. Chest imaging shows patchy infiltrative shadows, consolidation shadows in lobes or segments, ground glass shadows, or interstitial changes with or without pleural effusion; ③ Perform bronchoscopy and lavage; ④ The clinical strains isolated from the lavage solution were identified as *Haemophilus influenzae* by mass spectrometry (Merieux VITEK MS, France). Exclusion criteria: ① Exclude diseases such as pulmonary tuberculosis, lung tumors, noninfectious pulmonary interstitial diseases, pulmonary edema, pulmonary embolism, pulmonary vasculitis, etc.; ② Unable to obtain informed consent. The healthy control group consisted of children who underwent physical examinations in the Department of Child Health in Shenzhen Children’s Hospital. Inclusion criteria: ① Age range from 1 month to 14 years old; ② No respiratory tract infection; ③ The clinical strain isolated from the nasopharynx was identified by mass spectrometry (Merieux VITEK MS, France) as *Haemophilus influenzae*. Exclusion criteria: ① Chronic underlying diseases of the nose or lung; ② Symptoms of respiratory tract infection within two weeks; ③ Unable to obtain informed consent. Bronchoalveolar lavage were performed on hospitalized children according to clinical purposes, and the bronchoalveolar lavage fluids were routinely cultured for common bacteria such as *Streptococcus pneumoniae*, *Haemophilus influenzae*, and *Staphylococcus aureus*. All isolated clinical strains were identified using a mass spectrometer (BioMérieux VITEK MS, France). Ethical approval for this study was obtained from the Ethical Committee of Shenzhen Children’s Hospital (Shenzhen, Guangdong Province, China) under registration number 2,016,013.

### Typing of *Haemophilus influenzae* strains

Total DNA was extracted from the *Haemophilus influenzae* selected from the sample bank, and PCR was performed to detect the capsule gene (*bexA*) of *Haemophilus influenzae* using *Haemophilus influenzae* B (ATCC10211) as a positive control. Agarose gel electrophoresis was used to observe the PCR products for the typing identification of *Haemophilus influenzae* strains.

### Detection of adherence of NTHi to pulmonary epithelial cells

The A549 cells was resuscitated and cultured in 24-well plates at a density of 2.0 × 10^4^ cells/well. Cells were cultured overnight at 5% CO_2_ and 37℃, and were then used for subsequent experiments on the adherence of NTHi.

NTHi strains isolated from different samples were divided into groups and their adherence abilities to A549 cells in 24-well plates were tested. The bacterial suspension of each strain was diluted to 1.0 × 107 CFU/ml, and 100 ul/well were inoculated into the 24-well plates. Three replicate wells were set up for each group, and PBS was used as blank control. The plates were incubated at 5% CO2 and 37℃ for one hour, washed with PBS three times to remove bacteria that did not adhere to the cells, and then lysed with 1% saponin. The cells were diluted according to the ratio and inoculated onto chocolate agar plates, which were cultured overnight at 5% CO2 and 37℃. The colonies were counted for CFU the next day.

### Statistical analysis

The adherence abilities of NTHi from healthy children’s nasopharynx, hospitalized patients’ sputum, and bronchoalveolar lavage fluids to A549 cells were compared between groups. The comparisons between three groups were performed using the Kruskal-Wallis test, while pairwise comparisons were conducted using the t-test (SPSS version 21.0).

## Results

### Strain grouping

From June 2019 to May 2022, a total of 126 *Haemophilus influenzae* strains were selected from the sample bank, including 44 strains cultured from the nasopharynx swabs of healthy children, 24 isolates cultured from sputum of inpatients with pulmonary infections, and 58 isolates cultured from bronchoalveolar lavage fluids of hospitalized patients with pulmonary infections.

### Typing of *Haemophilus influenzae* strains

**Fig. 1:**
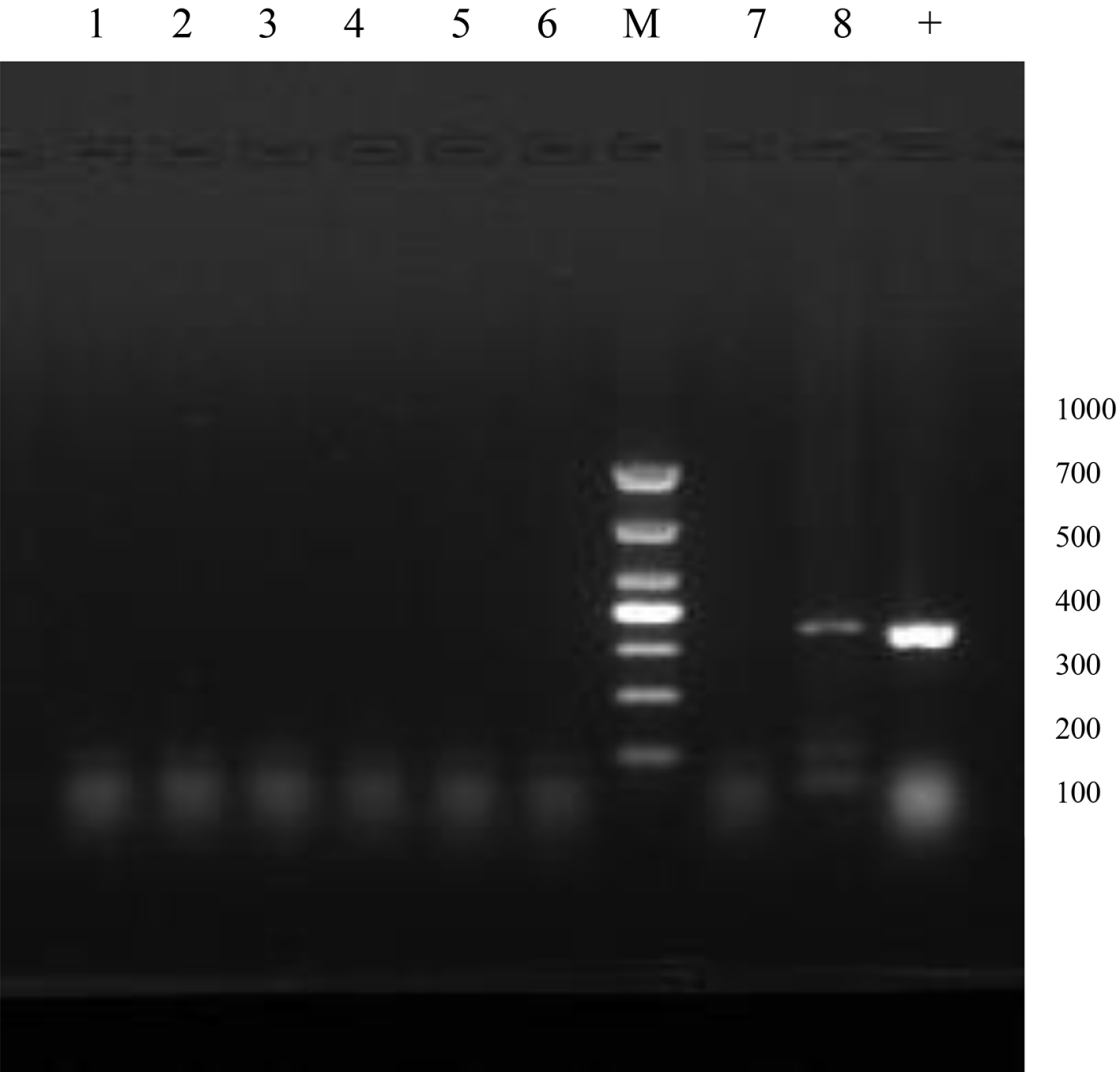
The typing results of *Haemophilus influenzae* strains (part of strains). The amplification product of the standard strain (+) contains the *bexA* gene, which is detected at 343 bp with a clear band. No target band is detected in most of the other strains, including sample No. 8, which is one of the positive samples. 1 and 2 are isolated strains from the nasopharynx of healthy children, 3, 4, and 5 are isolated strains from the sputum of children with lung infection, 6, 7, and 8 are isolated strains from the bronchoalveolar lavage fluid of children with acute lung infection, and M is a DNA marker

Using *Haemophilus influenzae B* (ATCC10211) as a positive control, total DNA was extracted from all clinical strains and standard strains, and PCR was performed to amplify the capsule gene (*bexA*). It was found that 125 strains were NTHi, and 1 strain isolated from bronchoalveolar lavage fluid was not included due to the presence of a capsule in this study (Fig. 1 and Supplementary Material 1).

### Adherence of NTHi to A549 cells

**Fig. 2:**
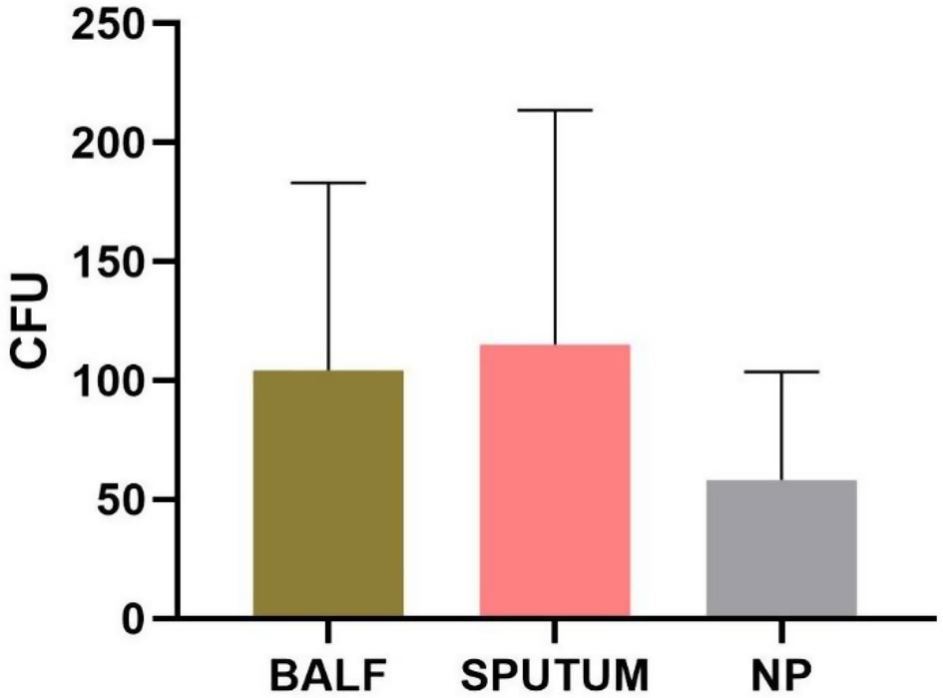
Adherence of three groups of nontypeable *Haemophilus influenzae* to A549 cells

The adherence of NTHi cultured from different sample sources to A549 cells was tested. The results showed that the mean adherence of 44 strains from healthy children’s nasopharynx swabs to A549 cells was 58.2 ± 45.3 CFU, while the mean adherence of 24 isolates from sputum was 115.1 ± 96.4 CFU. The mean adherence of 57 strains from bronchoalveolar lavage fluids was 104.3 ± 78.0 CFU (Fig. 2). There were significant differences between the three groups (F = 6.597, *P* = 0.002). The adherence of strains from sputum to A549 cells was significantly higher than that of isolates from healthy children’s nasopharynx swabs (t = 2.949, *P* = 0.004), and the adherence from bronchoalveolar lavage fluids to A549 cells was also significantly higher than that of isolates from healthy children’s nasopharynx (t = 3.221, *P* = 0.002). However, there was no significant difference between strains from sputum and bronchoalveolar lavage fluids (t = 0.522, *P* = 0.603).

## Discussion

Since the widespread use of the Haemophilus influenzae B conjugate vaccine, NTHi has become one of the major opportunistic pathogenic bacteria, including in otitis media, chronic obstructive pulmonary disease, and childhood pneumonia[12]. In this study, the majority of isolates were NTHi, and only one isolate from bronchoalveolar lavage fluid was typed, consistent with studies both domestically and internationally[1, 3, 13], indicating that the predominant strains in this region are NTHi.

The adherence of NTHi isolates from different populations and sites to A549 cells was tested. The strains from sputum or bronchoalveolar lavage fluids were more adherent to A549 cells than those isolated from healthy children’s nasopharynx swabs, consistent with isolates from otitis media and chronic obstructive pulmonary disease patients[14, 15]. It is possible that during infection, NTHi may migrate from the colonized nasopharyngeal region to the infected site, leading to changes adherence to epithelial cells to adapt to the new environment. HMW1A phase variation lowers adhesin expression, which controls an NTHi lifestyle switch from high epithelial invasiveness to lower invasion and higher biofilm formation[16]. The isolates from sputum and bronchoalveolar lavage fluids had similar adherence to pulmonary epithelial cells, indicating that during pulmonary infection, NTHi may undergo adaptive changes but further research is needed to confirm this. The study of the adherence of NTHi in otitis media and chronic obstructive pulmonary disease is currently a research hotspot [4, 13], and whether the adaptation mechanism and function mechanism in childhood pulmonary infections are consistent with otitis media or chronic obstructive pulmonary disease require further study.

In this study, there are some limitations: firstly, the control group was isolated from healthy children’s nasopharynx. The differences in isolate sites may have had an impact on the results. Secondly, the number of NTHi in this study was relatively small, and finally, the age range of children with NTHi sources studied was large. However, there was no significant difference in the adherence of isolates from sputum and bronchoalveolar lavage fluids to pulmonary epithelial cells, indicating that the adherence of isolates may only be related to changes in their own adherence or adaptive enhancement. In addition, the study of bacterial adherence only used traditional colony counting methods without validation at the gene or protein level of the isolate itself, which needs further exploration in future research.

### Electronic supplementary material

Below is the link to the electronic supplementary material.

Supplementary Material 1

## Data Availability

All data generated or analyzed during this study are included in this published article.
