# Supplementary figures and images for: Comparison of the adherence of nontypeable haemophilus influenzae to lung epithelial cells
Source: BMC Infect Dis. 2024 Feb 12;24:188. doi: 10.1186/s12879-024-09085-7 (PMC10863205; doi:10.1186/s12879-024-09085-7)

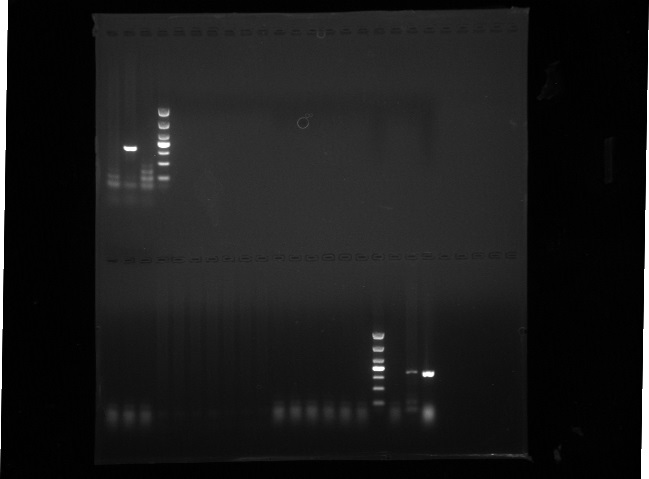

Supplement: Supplementary file 1 — Supplementary Material 1 [file 12879_2024_9085_MOESM1_ESM.jpg]
